# Reliability, Quality, and Readability of Freely Available Large Language Models for Clinical Decision Support in Perforated Peptic Ulcer: A Blinded, Multidimensional Study

**DOI:** 10.3390/diagnostics16203335

**Published:** 2026-10-14

**Authors:** Murat Coskun, Fatih Berke Inan, Bahaeddin Umur Aka, Alp Omer Canturk

**Affiliations:** 1 Department of General Surgery, Sakarya Training and Research Hospital, Sakarya 54100, Turkey; b.umur.aka@gmail.com; 2 Wallace H. Coulter Department of Biomedical Engineering, Georgia Institute of Technology, Atlanta, GA 30332, USA; finan3@gatech.edu; 3 Faculty of Medicine, Sakarya University, Sakarya 54100, Turkey; dr.omercanturk@gmail.com

**Keywords:** large language models, perforated peptic ulcer, emergency surgery, clinical decision support, artificial intelligence, DISCERN, GQS

## Abstract

**Background/Objectives**: Perforated peptic ulcer (PPU) is a common surgical emergency in which each hour before surgery increases mortality, and the decision is often made by a single surgeon on call. Large language models (LLMs) may offer rapid decision support, but their reliability in this setting is unknown. We compared five freely accessible LLMs in a blinded, multidimensional design. **Methods**: Twenty-four structured questions derived from the WSES 2020 guideline, covering the preoperative, intraoperative, and postoperative phases, were posed to the free versions of ChatGPT (GPT-5.3 Instant), Claude (Sonnet 4.6), DeepSeek-V3, Gemini (2.0 Flash), and Grok 3 during April and May 2026, yielding 120 responses. Two general surgeons blinded to model identity scored scientific reliability with the modified DISCERN instrument (mDISCERN, 8–40) as the primary measure and overall quality with the Global Quality Score (GQS, 1–5) as a secondary measure. Because every question was answered by all five models, the Friedman test was primary. **Results**: Models differed significantly in reliability (Friedman chi-square(4) = 71.35, *p* < 0.001, Kendall W = 0.743) and separated into two tiers: Claude (mDISCERN 37.83), Grok 3 (36.08), and DeepSeek-V3 (34.92) scored higher than ChatGPT (30.62) and Gemini (29.48). All between-tier comparisons were significant, whereas no within-tier comparison reached significance. Inter-rater agreement was moderate (mDISCERN ICC(2,k) = 0.647) and internal consistency high (Cronbach alpha 0.867 and 0.916). Reliability correlated positively with response length (rho = 0.717) and negatively with readability (rho = −0.452). **Conclusions**: Freely accessible LLMs vary substantially in reliability for PPU, so model choice has clinically meaningful consequences. This variation is specific to the models, versions, and access tier evaluated, and no model should be used for emergency surgical decision support without independent, setting-specific validation.

## 1. Introduction

Perforated peptic ulcer (PPU) remains the most frequently encountered high-mortality acute surgical emergency worldwide [1]. An aging population and increased use of nonsteroidal anti-inflammatory drugs have contributed to rising disease incidence [2]. Each hour of delay from diagnosis to surgery is associated with a 2.4% decrease in survival, and among patients in shock this delay corresponds to a 6% decrease in survival [3,4]. These findings underscore the importance of rapid decision-making in PPU. In elective procedures, the surgeon can discuss the decision at a multidisciplinary conference, consult the literature, and defer operation if necessary. In PPU, however, the decision is often made at night by a single surgeon on call. In this time-pressured setting, where rapid decision support is most needed and hours translate into measurable mortality, standardized and accurate decision-making is vital. The WSES and EAES provide algorithms for diagnosis and treatment [5,6], but the quality of evidence remains weak, and meta-analyses highlight persistent bias in this field [7]. This gap has driven development of validated risk-stratification tools such as Boey and PULP [8,9]. However, standardization and the desired clinical impact remain inadequately achieved.

This need has positioned large language models (LLMs), which process natural language with human-like fluency, as candidate tools for clinical decision support and surgical training. Their appeal is greatest precisely where structured guidance is scarce at the point of care, such as the emergency setting. Within surgery, potential applications in diagnostic support, triage, and patient communication are receiving increasing attention [10,11], and retrieval-augmented generation and the SurgeryLLM framework can integrate current guideline evidence into responses in real time [12,13]. Early work is encouraging: an LLM-generated appendicitis guideline showed acceptable consistency when appraised with the AGREE-S instrument [14,15]. This promise, however, is conditional on demonstrating reliability in the specific clinical task, because general-purpose competence does not transfer automatically to high-risk clinical decisions.

Several lines of evidence show why task-specific evaluation is essential. Medical LLMs can be vulnerable to data poisoning, and poisoned models perform identically to clean models on the open-source benchmarks routinely used to evaluate them [16]; general-purpose benchmarks may therefore miss harmful outputs in a specific clinical context. Consistent with this, the near-perfect scores LLMs achieve on medical licensing examinations do not reflect the complexity of real practice, and evaluation frameworks aligned with the clinical task itself have proven necessary [17]. Current models reach high accuracy in final diagnosis yet perform poorly in generating differential diagnoses and managing uncertainty, and they have not yet reached the competence required for safe clinical use [18]. Because most evaluations rely on static, single-pass responses, they also underestimate the errors that arise in dynamic diagnostic workflows [19,20].

These dimensions cannot be captured by a single overall score. Scientific reliability, educational value, and readability must instead be examined separately and in a procedure-specific manner, with attention to syntactic complexity, terminological richness, and information density [21]. Existing evaluations, however, are largely limited to single-scale or two-model comparisons [22] or to patient-oriented information [23]. No equivalent framework exists for emergency general surgery, which is structurally distinct from the elective setting because of its time constraints and limited access to consultation. A further limitation is that most evaluations use paid or flagship versions, whereas clinical utility often depends on freely accessible models, whose adequacy remains underexplored. Because general-purpose models have outperformed specialized commercial clinical tools, independent real-world evaluation is essential [24].

To address this gap, we conducted a procedure-specific, blinded, multidimensional evaluation of five current, freely accessible large language models in perforated peptic ulcer management based on guideline-derived evidence, spanning preoperative, intraoperative, and postoperative phases. Blinded general surgeons scored the models using objective systems that assessed readability and text complexity. To our knowledge, this is the first comprehensive, reproducible, and comparative evaluation of large language model performance in acute general surgery across multiple models. Moreover, it establishes a reproducible framework for selecting large language models for decision support in acute surgical contexts and delineates the evidence-based boundaries for integrating these tools into acute operative decision-making.

## 2. Materials and Methods

### 2.1. Study Aim and Design

This analytical cross-sectional study compared five current free large language models for scientific reliability, content quality, and readability using structured questions aligned with guidelines for the preoperative, intraoperative, and postoperative phases of perforated peptic ulcer management. The study was conducted during April and May 2026 and followed the STROBE guideline for design, data collection, and reporting.

### 2.2. Question Development and Content Validity

To establish a standardized testing foundation for LLMs, a 24-item question pool was created from high-level evidence sources. Questions were developed according to WSES and EAES guidelines and literature on validated perforated peptic ulcer risk scoring systems [5,6,8,9,25,26]. Items were designed to address perioperative risk stratification of perforated peptic ulcer, diagnostic algorithms, surgical technique selection, and postoperative complication management.

The content validity of the question set was evaluated using the Content Validity Index (CVI) method [27,28,29]. Three independent general surgeons with at least 10 years of experience rated each item on a 1–4 Likert scale across three dimensions: relevance, clarity, and applicability (1 = not relevant, 2 = somewhat relevant, 3 = quite relevant, 4 = highly relevant). Item-level validity (I-CVI) was calculated as the proportion of experts rating the item 3 or 4 divided by the total number of experts.

The acceptance threshold was determined according to the number of experts on the panel. With three members, a unanimity threshold (I-CVI = 1.00) was applied; items that did not achieve consensus were revised and reviewed iteratively until expert agreement was reached. This process yielded a finalized question set of 24 items: eight preoperative, eight intraoperative, and eight postoperative. The complete list of questions is presented in Table S2, and Table S1 provides a full inventory of evidence sources for each question. The three expert surgeons who participated in the content validity stage were excluded from the subsequent response-scoring phase.

### 2.3. Model Eligibility and Versions

Model selection followed established methodological frameworks for artificial intelligence comparisons in medical and surgical research [13,21]. Platforms were identified to meet the following criteria: public accessibility without institutional restrictions, stable interface and version traceability, fluent English communication in surgical contexts, and widespread adoption in academic and clinical communities. Only the freely accessible version of each platform was included as a deliberate and clinically justified design choice. Accordingly, the free versions of ChatGPT (GPT-5.3 Instant; OpenAI), Claude (Sonnet 4.6; Anthropic), DeepSeek-V3 (DeepSeek), Gemini (2.0 Flash; Google), and Grok 3 (xAI) were analysed. All responses were collected during a single evaluation window in April and May 2026, and the exact free-tier version available for each platform during this window is reported to support reproducibility. Because large language models are updated frequently, these findings apply to the specific versions and access tier evaluated here.

### 2.4. Response Collection

For each LLM, separate user accounts were created in separate browser sessions to prevent cross-platform interaction and IP overlap. All twenty-four configured questions were asked directly in English to eliminate potential translation bias. In each session, a standard opening prompt, ‘I am a general surgeon, please answer my questions accordingly’, was used to obtain expert-level response formatting.

To ensure reproducibility, all prompts were run through each model’s standard web-based user interface with default platform parameters; no manual adjustments were made to temperature, top-p, or token limits.

To limit the probabilistic response variability of large language models, the first generated response was recorded for each question; no regenerate, retry, or response-editing functions were used on any platform. Each question was posed in a separate session. Under this protocol, 24 questions were directed to five platforms, yielding 120 unique responses that were included in subsequent analyses. Raters scored each response from the complete original model output. For 11 of Claude’s longer responses, the text stored in the supplementary spreadsheet was shortened by the spreadsheet cell character limit; this truncation affected only the archived file and not the outputs presented to the raters, so scoring was based on the full responses.

### 2.5. Text Quality, Readability, and Structural Metrics

Responses were analysed according to multidimensional criteria summarized in Table 1, which assessed content accuracy, language clarity, and structural features.

The text quality was assessed using the eight-item modified DISCERN (mDISCERN) scale and the Global Quality Score (GQS) [30,31]. Both instruments appraise the reliability, completeness, source reporting, balance, and overall usefulness of health information. Neither adjudicates whether an individual clinical recommendation is factually correct or concordant with a specific guideline statement, and neither was designed to flag potentially harmful advice; the scores should therefore be read as measures of information quality rather than of clinical correctness.

The mDISCERN and GQS scores were independently assessed by two general surgeons uninvolved in the content validity phase: a newly qualified specialist and one with four years of specialist experience. Before assessment, both raters completed a calibration session using five pilot responses to standardize scale application.

Blinding was implemented with particular care given its critical role in ensuring methodological integrity. Platform identities were completely masked with randomly assigned A–E labels, randomized independently for each question. A third researcher, who did not participate in scoring, generated and retained the randomization key. Both raters, including the study executor, remained fully blinded and had no access to the key until scoring was complete. The key was opened only after scoring was finished and data were unblinded.

Readability and text structure were assessed using word count, sentence count, Flesch Reading Ease Score (FRES), Flesch–Kincaid Grade Level (FKGL), Gunning Fog Index, Simple Measure of Gobbledygook (SMOG), Coleman–Liau Index (CLI), and lexical density [32,33] (Table 1).

In readability calculation, a key technical issue arising from the structural nature of LLM output required resolution. Large language models commonly format their responses using bullet points, headings, and lists, elements that lack sentence-final punctuation. In standard readability algorithms, this causes multiple bullet points to be treated as a single sentence, producing invalid values in indices based on word-to-sentence ratios (FRES, FKGL, Fog, SMOG, CLI). To address this, all 120 responses underwent the same preprocessing pipeline before calculation. Markdown formatting elements were removed, bullet points and headings were designated as sentence boundaries, and text was normalized. The process was applied uniformly across each model and response without distinction. Readability indices were calculated from normalized text in a Python 3.12 environment using the textstat library. Lexical density, being independent of sentence boundaries, was unaffected by this procedure. The complete question list and 120 raw responses obtained from five models are presented in Table S2.

### 2.6. Statistical Analysis

The study used a paired design because all five models received the same 24 questions; each question therefore functioned as a matched block across models. The Friedman test for related samples was the primary method for comparing models, with agreement across questions quantified using Kendall W and pairwise comparisons made with Bonferroni adjustment. The Kruskal–Wallis test, which treats the models as independent groups, was reported as a supporting analysis, with effect size expressed as epsilon squared (H/(n − 1)) and pairwise comparisons made using the Dunn–Bonferroni test. Distributions were examined within each model group using the Shapiro–Wilk test and reported descriptively. For the family of eight readability and text-structure outcomes, *p* values were adjusted with the Holm method. Pairwise associations were assessed with Spearman rho; because the 120 responses are nested within 24 questions, the associations were repeated in a question-aware sensitivity analysis using within-question rank associations and cluster-level permutation *p* values. Inter-rater agreement was assessed with the intraclass correlation coefficient, and the internal consistency of the eight mDISCERN items with Cronbach alpha and item analysis for each rater. Because scientific reliability (mDISCERN) and content quality (GQS) differed in inter-rater agreement, mDISCERN was designated the primary quality measure and GQS a secondary, supporting measure. Tests were two-sided with alpha set at 0.05. All inferential analyses reported here were performed in SPSS 24.0 by an author with statistical expertise who took no part in question development, response collection, or scoring, and who received the dataset after scoring was complete and the blinding key had been opened.

The intraclass correlation coefficient was specified as ICC(2,k) based on absolute agreement in a two-way random effects model and ICC(3,k) based on consistency in a two-way mixed effects model, with confidence intervals calculated by the McGraw and Wong method. ICC values were interpreted as poor (<0.50), moderate (0.50 to 0.75), good (0.75 to 0.90), and excellent (>0.90) [34]. Agreement between the two raters was characterised further with a Bland–Altman approach, reporting the mean between-rater difference, the standard deviation of the differences, and the 95% limits of agreement. For GQS, which each rater recorded as an ordinal category from 1 to 5, ordinal agreement beyond chance was quantified with the quadratic weighted kappa. Internal consistency of the eight mDISCERN items was quantified with Cronbach alpha, reported separately for each rater with 95% confidence intervals and accompanied by item analysis reporting corrected item-total correlations and alpha if item deleted.

Robustness was assessed through prespecified sensitivity analyses. Responses whose archived text had been shortened were excluded, first row-wise and then by restricting the analysis to the 13 questions for which no response was affected, which preserves the paired structure. Each rater’s scores were analysed separately, and each of the three surgical phases was analysed independently.

Readability indices were computed in Python 3.12 as described in Section 2.5; all inferential analyses reported in this article were performed in SPSS. No a priori power calculation was undertaken, and observed post-hoc power is not reported because effect sizes, adjusted *p* values, and confidence intervals are more informative for a design of this kind.

### 2.7. Ethical Considerations

This study involved no direct application or intervention on human or animal subjects; the evaluated content consisted of texts generated by artificial intelligence, and no patient data were used. Accordingly, ethical board approval was not required. Throughout the study, potential ethical concerns regarding the use of artificial intelligence-supported tools in medical education were considered, and data privacy, impartiality, and scientific ethics principles were carefully observed in accordance with the fundamental principles of the Declaration of Helsinki.

## 3. Results

### 3.1. General Findings and Dataset

**Figure 1:**
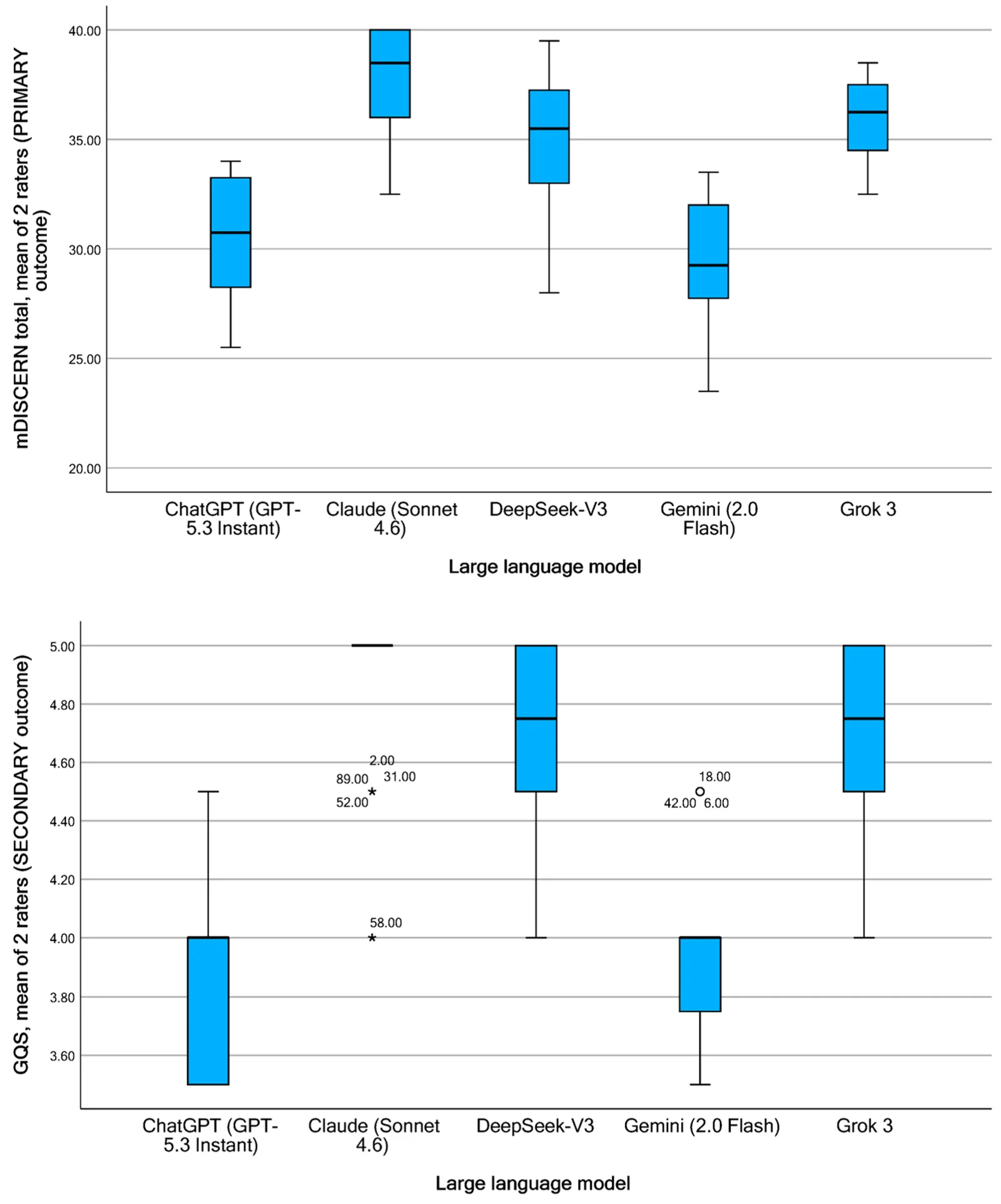
Distribution of modified DISCERN (mDISCERN, upper panel) and Global Quality Score (GQS, lower panel) values across the five large language models. Boxes show the interquartile range and the median; whiskers extend to the most extreme values within 1.5 interquartile ranges; circles and asterisks mark mild and extreme outliers, labelled with the case number. Output generated in SPSS.

Five major language models were administered 24 structured questions, yielding 120 unique responses that underwent analysis. For each model, 24 responses were evaluated across three surgical phases: eight preoperative, eight intraoperative, and eight postoperative. The dataset contained no missing observations. Because the paired design assigned the same 24 questions to every model, the Friedman test was used as the primary comparison, with the Kruskal–Wallis test reported as a supporting distribution-free analysis. Scientific reliability, measured by mDISCERN, was the primary quality outcome, and GQS served as a secondary measure. The distribution of scores across models is shown in Figure 1.

### 3.2. Scientific Reliability (mDISCERN)

A significant difference in scientific reliability was found among models. Claude achieved the highest mean mDISCERN score (37.83), followed by Grok 3 (36.08) and DeepSeek-V3 (34.92). ChatGPT (30.62) and Gemini (29.48) recorded the lowest scores (Table 2).

The Friedman test showed a significant difference in scientific reliability among models (chi-square(4) = 71.35, *p* < 0.001, Kendall W = 0.743). Post-hoc pairwise comparisons showed that the models separated into two performance tiers. All comparisons between the higher tier (Claude, Grok 3, and DeepSeek-V3) and the lower tier (ChatGPT and Gemini) were significant (*p* < 0.01), whereas no comparison within either tier reached significance after adjustment for multiple comparisons. The separation corresponded to a clear gap in mean mDISCERN scores, from 34.92 in the lowest-scoring higher-tier model to 30.62 in the highest-scoring lower-tier model. The supporting Kruskal–Wallis analysis was consistent (H(4) = 76.28, *p* < 0.001, epsilon-squared = 0.641).

### 3.3. Content Quality (GQS)

A statistically significant difference between models was found for GQS. Rankings largely overlapped with mDISCERN, with Claude showing the highest GQS (4.88). However, the lower-performing models diverged: ChatGPT scored lowest on GQS (3.85), while Gemini ranked slightly higher (3.96). Thus, ChatGPT demonstrated the lowest content quality, whereas Gemini exhibited the lowest scientific reliability.

GQS, analysed as a secondary measure, followed the same two-tier pattern. The Friedman test was significant (chi-square(4) = 73.27, *p* < 0.001, Kendall W = 0.763), and post-hoc comparisons between the higher tier (Claude, Grok 3, DeepSeek-V3) and the lower tier (ChatGPT, Gemini) were significant (*p* < 0.001), while no within-tier comparison reached significance. The supporting Kruskal–Wallis analysis was consistent (H(4) = 75.72, *p* < 0.001, epsilon-squared = 0.636).

### 3.4. Inter-Rater Reliability

Agreement between the two independent blinded general surgeons’ scores was moderate for both measures. For mDISCERN, the ICC(2,k) for absolute agreement was 0.647 (95% CI 0.494 to 0.753) and the ICC(3,k) for consistency was 0.654 (95% CI 0.504 to 0.759). For GQS, the corresponding values were 0.507 (95% CI 0.253 to 0.669) and 0.555 (95% CI 0.362 to 0.690). Bland–Altman analysis indicated that disagreement between the raters was largely random rather than systematic. The mean difference was −0.99 points for mDISCERN (SD of differences 4.80; 95% limits of agreement −10.41 to 8.43) and −0.33 points for GQS (SD of differences 0.70; 95% limits of agreement −1.71 to 1.04); both mean differences are small relative to the respective scale ranges, although the limits of agreement for mDISCERN were wide. For GQS, the quadratic weighted kappa was 0.337, indicating limited ordinal agreement beyond chance. The internal consistency of the eight mDISCERN items was high for both raters (Cronbach alpha 0.867, 95% CI 0.828 to 0.900, and 0.916, 95% CI 0.891 to 0.937), and no item improved the scale when removed.

### 3.5. Readability and Text Structure

Readability differed significantly across models for every index, and all comparisons remained significant after Holm correction for multiple testing. Gemini produced the most readable responses according to Flesch Reading Ease (26.5), while Claude produced the least readable, reflecting the most technical and dense text (9.3). Flesch–Kincaid grade levels ranged from 12.6 to 15.0 across models, indicating that every model wrote at a level suited to health professionals rather than the general public (Table 3).

Response length differed statistically across models. Claude produced the longest responses, with an average of 2998 words, whereas Gemini (508 words) and ChatGPT (514 words) provided the shortest outputs. Lexical density also varied significantly: Claude and ChatGPT showed the highest density at approximately 82%, while other models ranged from 70–75%.

### 3.6. Quality, Length, and Readability

The two quality measures were strongly correlated (Spearman rho = 0.886, 95% CI 0.845 to 0.914, *p* < 0.001). Scientific reliability increased with response length (mDISCERN with word count rho = 0.717, 95% CI 0.617 to 0.792, *p* < 0.001) and decreased as text became easier to read (mDISCERN with Flesch Reading Ease rho = −0.452, 95% CI −0.588 to −0.293, *p* < 0.001). The association with lexical density was weak (rho = 0.270, 95% CI 0.089 to 0.438, *p* = 0.003). Because the 120 responses are nested within 24 questions, these pooled coefficients were repeated in a question-aware sensitivity analysis; the associations remained in the same direction and significant (mDISCERN with word count 0.779 and mDISCERN with Flesch Reading Ease −0.390, both *p* < 0.001 by cluster-level permutation).

### 3.7. Robustness Analyses

Model differences were robust across sensitivity analyses. First, excluding the 11 Claude responses whose archived text was shortened by spreadsheet cell limits left the difference significant (Kruskal–Wallis H = 67.13, *p* < 0.001, *n* = 109), and an analysis restricted to the 13 questions with no truncated response, which preserves the paired structure, gave the same result (Friedman chi-square(4) = 36.68, *p* < 0.001, Kendall W = 0.705). Second, the difference remained significant within each surgical phase analysed separately (Friedman chi-square(4) = 24.08, 23.46, and 26.29 for the preoperative, intraoperative, and postoperative sets; all *p* < 0.001). Third, the difference was significant when each rater’s scores were analysed alone (Friedman chi-square(4) = 47.32 and 80.10; both *p* < 0.001). The supporting Kruskal–Wallis analysis agreed with the primary Friedman result for both mDISCERN and GQS.

## 4. Discussion

Three main findings emerge from this evaluation. First, the models differed substantially and significantly in both scientific reliability and content quality, with a large effect size (Kendall W = 0.743 for mDISCERN), and they separated into two performance tiers. Second, Claude achieved the highest performance, followed by Grok 3 and DeepSeek-V3, while ChatGPT and Gemini formed a statistically separate lower tier; the gap between the two tiers spanned more than four mDISCERN points, whereas differences within each tier were not statistically detectable. Third, an inverse relationship emerged between quality and readability, with the most reliable responses being the densest and most difficult to read. Collectively, these findings show that the choice among freely available models has clinically meaningful consequences for the reliability of decision support in the acute surgical setting.

### 4.1. Comparison of Findings with Elective Surgery Data

Our results differ from elective surgery comparisons in the literature. In the methodologically closest study, Grok achieved the highest mean mDISCERN score, while Gemini demonstrated the lowest [21]. In our emergency surgery cohort, however, Claude received the highest score, with Grok 3 ranking second; Gemini attained the lowest mDISCERN score, while ChatGPT had the lowest GQS score. Several factors differ between the two studies at the same time, including the clinical procedure, the specific model versions, and the use of free rather than flagship tiers, so these differences cannot be attributed to procedural context alone. Taken together, they carry an important methodological caution: model rankings obtained under one combination of procedure, version, and access tier cannot be assumed to transfer to another.

This divergence is consistent with the premise that emergency and elective surgery differ not only in subject matter but also in problem structure. Questions about perforated peptic ulcer encompass resuscitation under time pressure, the limits of non-operative management, damage control, and giant ulcer, scenarios that demand a different knowledge base and depth of reasoning from elective cholecystectomy guidelines. A model intended for decision support in emergency surgery should therefore be evaluated within the emergency surgery context rather than assumed to perform comparably on the basis of elective data.

### 4.2. Quality, Length, and Readability: Interpretation

Scientific reliability shows a moderate-to-strong positive relationship with response length, whereas readability exhibits a negative relationship, establishing a consistent pattern in how quality emerges. Claude, which achieved the highest reliability score, produced the longest responses, averaging approximately 3000 words, and the lowest Flesch readability score. Gemini delivered the most readable responses but produced the shortest and lowest-reliability answers. The weak relationship between quality and lexical density suggests that high quality correlates more with the volume and depth of clinical content covered than with word-level density alone. This pattern aligns with findings from the elective study, which reported that quality relates to terminological richness rather than syntactic complexity [21].

This relationship carries two clinical implications. Longer, more detailed responses may better satisfy the evidence depth required for urgent decision-making; conversely, all models maintained a Flesch–Kincaid grade level between 12.6 and 15.0, indicating that generated content suits healthcare professionals rather than average patients. For surgeon decision support, this readability level poses no limitation; however, it demonstrates that the same output cannot serve directly for patient-facing information.

Because mDISCERN rises with response length, the differences between models could in principle reflect verbosity rather than reliability. Two features of the data argue against that reading. First, the ordering of the models by length and by reliability is not the same: ranked by length they run Claude (2998 words), DeepSeek-V3 (1137), Grok 3 (725), ChatGPT (514) and Gemini (508), whereas ranked by mDISCERN they run Claude (37.83), Grok 3 (36.08), DeepSeek-V3 (34.92), ChatGPT (30.62) and Gemini (29.48). Grok 3 scored higher than DeepSeek-V3 while producing about a third fewer words, and ChatGPT and Gemini, whose mean lengths differ by six words, still differ in mDISCERN. Second, length and model identity are not separable in this design, because length is largely a property of the model rather than a free variable. Adjusting the comparison for word count would remove part of the model effect by construction, and the adjusted estimate would then be difficult to interpret. We therefore report the association and the ordering together and did not fit a length-adjusted model. The question would be settled better by a design in which response length is constrained by the prompt, so that length varies independently of model identity, than by a statistical adjustment applied after the fact.

A practical tension follows from the relationship between length and reliability. The most reliable model produced responses averaging about 3000 words, which is difficult to read when the operative decision cannot wait. The dissociation just described points to a way around this: because near-top reliability did not require the longest output, a shorter higher-tier model may be the more sensible choice where reading time is the binding constraint. A surgeon using a long-form model can also ask it for a brief structured summary before consulting the full answer, although we did not test summary-first prompting and its effect on reliability is unknown.

### 4.3. Clinical Reliability and Accessibility of Free Models

The most important finding is that success on general-purpose benchmarks does not guarantee reliability in a specific clinical task. Among the same free versions, a higher-reliability tier coexisted with a distinctly lower-reliability tier, so the choice of model directly affects the quality of clinical decision support in this setting. This matters clinically because a benchmark-strong model may still generate unreliable emergency guidance. Medical models can be vulnerable to data poisoning while performing identically to clean models on standard benchmarks [16]. Licensing-exam performance does not reflect real clinical complexity [17], and current models remain weak in differential diagnosis and uncertainty management [18]. Task-specific, procedure-level evaluation is therefore a prerequisite for safe use rather than an optional refinement.

The evaluation of freely accessible versions is a deliberate contribution of this study and increases the relevance of the findings to everyday practice, where cost and access often determine which tool a clinician actually uses. A free model has been reported to match a paid model when answering common patient questions about a surgical procedure [22], and general-purpose models can outperform specialized commercial clinical tools [24]. Our data extend this literature by showing clinically meaningful variation in reliability among free models applied to a single emergency procedure, which reinforces the need for independent, procedure-specific evaluation before any free tool is adopted for acute surgical decision support.

### 4.4. Clinical and Educational Implications

Perforated peptic ulcer management is time-critical, with each hour of surgical delay associated with measurable increases in mortality [3,4]. This urgency simultaneously heightens the demand for rapid, reliable decision support and amplifies the consequences of erroneous outputs. Our findings show that a well-selected model can deliver high-confidence answers to most WSES-based questions, yet this capability varies substantially across models. Consequently, integration of these tools into emergency surgical decision support cannot be recommended without prior model-level evaluation. Consistent with the broader recognition that artificial intelligence applications in surgery remain in an early stage and require careful evaluation frameworks [10], our study provides empirical evidence specific to emergency surgery that contributes to that effort.

What such an evaluation framework should contain can be stated concretely on the basis of our findings. Before a freely accessible model is used for decision support in a given procedure, we suggest four steps. The first is to assemble a guideline-anchored question set that covers the whole care pathway and to confirm its content validity. The second is to collect responses from the candidate models under a fixed prompting protocol. The third is to score them blinded, with at least two independent raters and a reliability estimate reported alongside the scores. The fourth is to compare models using a method matched to the paired structure of the data, before any model is adopted. Each step is reproducible with the materials provided here, and the same procedure can be repeated whenever a candidate model changes.

A single evaluation is unlikely to remain valid for long. Large language models are revised continuously, and a version change can alter output quality without any visible change to the user interface, so performance established at one time point may drift. Institutions considering deployment therefore need a way to detect this drift. The exact model version and access tier in clinical use should be recorded. A short fixed benchmark should be repeated whenever the provider releases a new version, and a fall in benchmark performance should trigger re-evaluation before continued use. A follow-up evaluation was outside the scope of the present study, but periodic revalidation of this kind, rather than one-time appraisal, is the realistic basis for sustained clinical use of these tools.

Selecting a model and monitoring it both assume that one model must be chosen. The two-tier separation raises a further possibility, namely that models might be combined rather than chosen between. Strategies such as majority voting across models, weighting each model by its measured reliability, or multi-agent deliberation in which models critique one another could in principle raise reliability above that of any single free model, and the spread observed here suggests room for such gains. Our design cannot test this directly, because mDISCERN and GQS appraise the quality and reliability of a free-text answer rather than scoring it against one correct option, so there is no discrete output on which votes could be counted. Evaluating ensemble approaches would require a different design, with a predefined reference standard for each question and an explicit rule for resolving disagreement between models, and we regard this as a promising direction for subsequent studies.

A further consequence of the present framing is that all five models were evaluated as stand-alone information sources, whereas their realistic clinical role is as an adjunct to surgical judgement rather than a substitute for it. Evidence from other specialties indicates that the combination can outperform either component on its own. In skin cancer recognition, clinicians supported by artificial intelligence were more accurate than either the clinicians or the algorithm alone [35]. A randomised trial of clinician–AI collaborative diagnostic workflows has since begun to examine how that interaction should be structured [36]. Our design cannot speak to this, because no surgeon arm was included. For perforated peptic ulcer, the decisive question is therefore not only which model is most reliable, but whether a surgeon assisted by a model reaches better decisions than either alone, and what that assistance costs in time when the operative decision cannot wait. A three-arm comparison of surgeon alone, model alone, and surgeon with model, conducted under realistic time pressure, would test the collaborative framing directly and is the study we regard as following most naturally from these results.

### 4.5. Strengths and Limitations

The study’s principal strengths include a structured question set derived from guidelines and validated for content validity, blinded and randomized scoring, reliability analysis with two independent raters, high internal consistency of the mDISCERN items, and a statistical approach matched to the paired design. The inferential analyses were carried out independently of question development, response collection, and scoring, as set out in Section 2.6. Robustness was further supported by sensitivity analyses that excluded truncated responses, preserved the paired structure using only questions with complete responses, examined each phase and each rater separately, and repeated the associations with a question-aware, cluster-level permutation approach.

The moderate agreement between the two raters deserves closer examination. Bland–Altman analysis showed no meaningful systematic bias between them, so the residual disagreement was mainly random scatter; the limits of agreement for mDISCERN were nonetheless wide, and a larger rater panel would be required to narrow them. The difference in agreement between the two instruments is also structurally explicable. mDISCERN averages eight separately rated items, so idiosyncrasies of an individual rater partly cancel, whereas GQS rests on a single global judgement per response with no internal averaging. Its confidence interval extended into the poor range and its quadratic weighted kappa was 0.337, so designating GQS as secondary rather than co-primary keeps the principal conclusions on the more reliable instrument.

Two observations limit the practical consequences of this disagreement. The model-level differences did not depend on which rater was analysed: when each rater’s scores were examined alone, the difference between models remained significant (Friedman chi-square(4) = 47.32 and 80.10, both *p* < 0.001) and the two-tier structure was preserved. The disagreement therefore affects the precision of the score given to an individual response rather than the ordering of the models. The clinical implication is that a single reader’s appraisal of one answer carries appreciable uncertainty, so a model should be chosen on aggregate performance across a structured question set rather than on impressions formed from a handful of responses. A larger panel of experienced raters with formal adjudication of discrepant scores would narrow the limits of agreement, and we regard it as a necessary component of any confirmatory study. It was not feasible here, because it would have required a new blinded scoring round after the blinding key had been opened.

The study has several limitations. First, the evaluation was conducted at a single center by two blinded general surgeons, and assessment scales such as mDISCERN and GQS introduce subjective elements. Agreement was moderate rather than high, and it was lower for GQS than for mDISCERN. This is why GQS was treated as a secondary measure. Second, the absence of significant differences within each performance tier should not be read as evidence that those models are equivalent; no within-tier difference was detected after adjustment for multiple comparisons, and the sample provides less precision for separating models of similar performance. Third, large language models undergo rapid updates; the versions evaluated here represent a specific time period, and results must be revalidated as models evolve; the monitoring approach we suggest for this is set out in Section 4.4. Fourth, responses were collected in a static manner; the iterative and interactive nature of real clinical practice may generate errors that such evaluations cannot detect [20]. Only the first generated response was analysed for each question, which standardised the comparison across models but left the within-model variability of repeated runs unmeasured. Repeated independent runs of the same question would be required to establish response stability and reproducibility, and we did not perform them because the protocol deliberately fixed a single first-pass response to keep the paired comparison identical across platforms. Fifth, no a priori power calculation was performed; the analysis was powered in practice by the paired design and the large observed effects, but small differences between closely performing models may have gone undetected. Sixth, evaluation was restricted to English-language responses, and performance across other languages remains unknown. Finally, although mDISCERN and GQS assess content quality and reliability, they do not directly measure the factual accuracy of individual clinical recommendations or their effect on patient outcomes. An item-level assessment of factual and guideline concordance, with explicit adjudication of potentially harmful recommendations, would address this directly and is in our view the single most important next step for this high-risk setting. It could not be added here, because it requires a fresh scoring round against individual guideline statements by raters blinded to model identity, and the blinding key had already been opened for the present analysis. These dimensions warrant investigation through future prospective and interactive study designs.

## 5. Conclusions

This study provides, to our knowledge, the first procedure-specific, blinded, and multidimensional evaluation of five freely accessible large language models for perforated peptic ulcer, a time-critical emergency. Models differed significantly in scientific reliability and content quality and separated into a higher-performing tier (Claude, Grok 3, DeepSeek-V3) and a lower-performing tier (ChatGPT, Gemini). The mDISCERN instrument behaved consistently in this setting, with high internal consistency across its eight items and moderate agreement between two independent blinded raters. Two features of the observed quality were clinically relevant: reliability tracked with information volume and terminological depth rather than easy readability, and the most reliable answers required a healthcare-professional reading level.

These findings carry a practical implication for how such tools should be adopted. Because model performance depended on the specific combination of procedure, version, and access tier evaluated, rankings obtained in one context should not be assumed to hold in another, and general-purpose benchmark standing is not a sufficient basis for clinical selection.

A carefully selected, freely accessible large language model may serve as a useful adjunct for emergency surgical decision support, but none can be recommended for this purpose without prior model-level, procedure-specific evaluation. By providing reproducible criteria for such evaluation, set out as a four-step procedure in Section 4.4, this study offers a practical framework for the safe and evidence-based integration of these tools into acute surgical care. Prospective, interactive studies are the next step.

## Figures and Tables

**Table 1:** Definition and computation of the quality, readability, and structural metrics.

Metric	Definition/Item Content	Computation	Interpretation
mDISCERN	Eight items: (1) explicit aims, (2) relevance to the question, (3) sources cited, (4) date of information, (5) balance and objectivity, (6) additional risks addressed, (7) areas of uncertainty, (8) overall quality	Sum of item scores (each 1–5); total range 8–40	Higher scores indicate greater scientific reliability (range 8 to 40).
GQS	Single item: “Overall, how useful is this response for a surgeon needing practical, evidence-based guidance?” (1 = very poor, 5 = excellent)	Direct rating, 1–5	Higher scores indicate higher overall quality (range 1 to 5).
FRES	Flesch Reading Ease Score	206.835 − 1.015 × (words/sentence) − 84.6 × (syllables/word)	0–100; higher = easier to read
FKGL	Flesch–Kincaid Grade Level	0.39 × (words/sentence) + 11.8 × (syllables/word) − 15.59	US school grade level
Fog	Gunning Fog Index	0.4 × [(words/sentence) + 100 × (complex words/word)]	US school grade level
SMOG	Simple Measure of Gobbledygook	1.043 × √(polysyllables × 30/sentences) + 3.1291	US school grade level
CLI	Coleman-Liau Index	0.0588 × (letters per 100 words) − 0.296 × (sentences per 100 words) − 15.8	US school grade level
Lexical density	Proportion of content (lexical) words	(content words/total words) × 100	%; higher = more information-dense text

mDISCERN, modified DISCERN instrument; GQS, Global Quality Score; FRES, Flesch Reading Ease Score; FKGL, Flesch–Kincaid Grade Level; SMOG, Simple Measure of Gobbledygook; CLI, Coleman–Liau Index. Readability indices were computed after sentence-boundary normalization of markdown-formatted model output (see Section 2). Quality scores are the mean of two independent, blinded general surgeons.

**Table 2:** Reliability and quality scores of the five large language models, with global comparison.

Model	mDISCERN Mean ± SD	mDISCERN Median (IQR)	GQS Mean ± SD	GQS Median
Claude	37.83 ± 2.33	38.50 (36.00–40.00)	4.88 ± 0.27	5.00
Grok 3	36.08 ± 1.77	36.25 (34.50–37.50)	4.71 ± 0.33	4.75
DeepSeek-V3	34.92 ± 3.05	35.50 (32.75–37.38)	4.65 ± 0.40	4.75
ChatGPT	30.62 ± 2.72	30.75 (28.12–33.38)	3.85 ± 0.28	4.00
Gemini	29.48 ± 2.71	29.25 (27.38–32.00)	3.96 ± 0.33	4.00

Values are per model across 24 responses. Models are ordered by descending mean mDISCERN. Friedman test (primary, paired design): mDISCERN chi-square(4) = 71.35, *p* < 0.001, Kendall W = 0.743; GQS chi-square(4) = 73.27, *p* < 0.001, Kendall W = 0.763. Kruskal–Wallis test (supporting): mDISCERN H(4) = 76.28, *p* < 0.001, epsilon-squared = 0.641; GQS H(4) = 75.72, *p* < 0.001, epsilon-squared = 0.636. Post-hoc pairwise comparisons with Bonferroni adjustment identified a higher-performing tier (Claude, Grok 3, DeepSeek-V3) and a lower-performing tier (ChatGPT, Gemini); all between-tier comparisons were significant (*p* < 0.01), whereas no within-tier comparison reached significance. mDISCERN, modified DISCERN instrument; GQS, Global Quality Score; SD, standard deviation; IQR, interquartile range.

**Table 3:** Readability and text-structure metrics of the five large language models.

Model	Word Count Mean ± SD	FRES Mean ± SD	FKGL	Fog	SMOG	CLI	Lexical Density (%)
Claude	2998 ± 1081	9.3 ± 7.6	14.0	17.2	12.8	18.6	81.9
Grok 3	725 ± 87	13.6 ± 7.4	15.0	17.8	15.2	18.2	74.5
DeepSeek-V3	1137 ± 328	16.9 ± 11.4	14.0	17.1	14.2	17.4	70.7
ChatGPT	514 ± 42	14.4 ± 9.5	12.9	15.8	11.6	16.9	81.9
Gemini	508 ± 56	26.5 ± 7.7	12.6	15.5	13.5	15.4	70.2

Values are the mean per model across 24 responses. Because the metrics are paired by question, models were compared with the Friedman test; the family comprised eight readability and text-structure outcomes, and all eight remained significant after Holm correction (word count chi-square(4) = 84.70, Kendall W = 0.882; sentence count 86.77, W = 0.904; FRES 41.03, W = 0.427; FKGL 44.83, W = 0.467; Fog 56.15, W = 0.585; SMOG 81.70, W = 0.851; Coleman–Liau 47.96, W = 0.500; lexical density 83.40, W = 0.869; all adjusted *p* < 0.001). Sentence count was tested as part of this family but is not tabulated; per-model values are provided in Data S1. Higher FRES indicates easier reading; higher FKGL, Fog, SMOG, and CLI indicate text requiring a more advanced education level. FRES, Flesch Reading Ease Score; FKGL, Flesch–Kincaid Grade Level; SMOG, Simple Measure of Gobbledygook; CLI, Coleman–Liau Index; SD, standard deviation.

## Data Availability

All data supporting the findings of this study are provided in the article and in the Supplementary Materials (Tables S1 and S2, and Data S1). Data S1 contains the item-level dataset with the scores of both raters for all 120 responses, the summary tables of the independent SPSS analysis, and the analysis syntax, so that every reported statistic can be recomputed from the raw scores. Further information is available from the corresponding author on reasonable request.

## Supplementary Materials

The following supporting information can be downloaded at: https://www.mdpi.com/article/10.3390/diagnostics16203335/s1. Table S1: Full inventory of the 24 structured questions with the evidence source for each; Table S2: Complete question set with the 120 raw model responses; Data S1: Statistical workbook containing the item-level dataset with the scores of both raters for all 120 responses, the summary tables of the independent SPSS analysis, and the analysis syntax.

## Abbreviations

The following abbreviations are used in this manuscript:

PPU	perforated peptic ulcer
LLM	large language model
mDISCERN	modified DISCERN instrument
GQS	Global Quality Score
ICC	intraclass correlation coefficient
CVI	Content Validity Index
FRES	Flesch Reading Ease Score
FKGL	Flesch–Kincaid Grade Level
SMOG	Simple Measure of Gobbledygook
CLI	Coleman–Liau Index
WSES	World Society of Emergency Surgery
EAES	European Association for Endoscopic Surgery
